# Dissecting tumor microenvironment heterogeneity in syngeneic mouse models: insights on cancer-associated fibroblast phenotypes shaped by infiltrating T cells

**DOI:** 10.3389/fimmu.2023.1320614

**Published:** 2024-01-08

**Authors:** Marco Carretta, Marie-Louise Thorseth, Aimilia Schina, Dennis Alexander Agardy, Astrid Zedlitz Johansen, Kevin James Baker, Shawez Khan, Anne Mette Askehøj Rømer, Klaire Yixin Fjæstad, Hannes Linder, Dorota Ewa Kuczek, Marco Donia, Lars Grøntved, Daniel Hargbøl Madsen

**Affiliations:** ^1^ National Center for Cancer Immune Therapy (CCIT-DK), Department of Oncology, Copenhagen University Hospital - Herlev and Gentofte, Herlev, Denmark; ^2^ Department of Biochemistry and Molecular Biology, University of Southern Denmark, Odense, Denmark; ^3^ Department of Immunology and Microbiology, University of Copenhagen, Copenhagen, Denmark

**Keywords:** tumor microenvironment, cancer-associated fibroblasts, syngeneic mouse cancer models, immunotherapy, immunosuppressive mechanisms, stroma, tissue stiffness, PD-L1

## Abstract

Murine syngeneic tumor models have been used extensively for cancer research for several decades and have been instrumental in driving the discovery and development of cancer immunotherapies. These tumor models are very simplistic cancer models, but recent reports have, however, indicated that the different inoculated cancer cell lines can lead to the formation of unique tumor microenvironments (TMEs). To gain more knowledge from studies based on syngeneic tumor models, it is essential to obtain an in-depth understanding of the cellular and molecular composition of the TME in the different models. Additionally, other parameters that are important for cancer progression, such as collagen content and mechanical tissue stiffness across syngeneic tumor models have not previously been reported. Here, we compare the TME of tumors derived from six common syngeneic tumor models. Using flow cytometry and transcriptomic analyses, we show that strikingly unique TMEs are formed by the different cancer cell lines. The differences are reflected as changes in abundance and phenotype of myeloid, lymphoid, and stromal cells in the tumors. Gene expression analyses support the different cellular composition of the TMEs and indicate that distinct immunosuppressive mechanisms are employed depending on the tumor model. Cancer-associated fibroblasts (CAFs) also acquire very different phenotypes across the tumor models. These differences include differential expression of genes encoding extracellular matrix (ECM) proteins, matrix metalloproteinases (MMPs), and immunosuppressive factors. The gene expression profiles suggest that CAFs can contribute to the formation of an immunosuppressive TME, and flow cytometry analyses show increased PD-L1 expression by CAFs in the immunogenic tumor models, MC38 and CT26. Comparison with CAF subsets identified in other studies shows that CAFs are skewed towards specific subsets depending on the model. In athymic mice lacking tumor-infiltrating cytotoxic T cells, CAFs express lower levels of PD-L1 and lower levels of fibroblast activation markers. Our data underscores that CAFs can be involved in the formation of an immunosuppressive TME.

## 1 Introduction

The past two decades have seen the rapid development of cancer therapies that directly involve the patient’s immune system (1, 2). The therapies are collectively termed immunotherapy and comprise a range of immunomodulating therapies that target different steps within the cancer immunity cycle with the goal of generating an anti-cancer response (3). These new immunotherapeutic approaches, such as immune checkpoint inhibitors (ICIs) targeting CTLA-4 or PD-1/PD-L1, have demonstrated clinical efficacy in a wide variety of solid tumors (4, 5) and have highlighted the critical role that the immune system plays in fighting cancer. However, only a minority of patients respond to these therapies and display long-term responses, while most patients do not respond or may develop resistance to the treatment (6, 7). The formation of an immunosuppressive tumor microenvironment (TME) has been suggested as a major obstacle to the successful outcome of immunotherapy and understanding how the TME influences anti-tumor immune activity currently represents a central theme in cancer immunotherapy research.

Studies of the immune composition of human tumors, have identified great heterogeneity between cancer types and between tumors of the same cancer type (8). A high level of tumor-infiltrating CD8^+^ T cells and Th1 cytokine expression, commonly defined as an immunogenic or ‘hot’ tumor, is generally associated with a good prognosis (9). Conversely, low T cell infiltration and the presence of suppressive myeloid cells contribute to the establishment of an immunologically ‘cold’ tumor, which is associated with poor prognosis (8). Some tumors display a third immune profile known as “immune-excluded”, characterized by the presence of immune cells confined to the stroma and lack of infiltration into the tumor nests (10). Immunosuppressive myeloid cells such as M2-like macrophages and myeloid-derived suppressor cells (MDSCs) have been shown to play major roles in the immune evasion of cancer cells (11, 12) and are also involved in remodeling of the surrounding extracellular matrix (ECM) (13–15).

Other mechanisms contributing to an immunosuppressive TME involve stromal cells such as cancer-associated fibroblasts (CAFs) (16, 17). These cells are the most abundant non-hematopoietic cells in the TME and cover a range of subsets and activation states rather than being a uniform cell type (18, 19). Several studies indicate that CAFs may have different origins, which might contribute to their dynamic heterogeneity and explain why common CAF-markers such as fibroblast-activation protein (FAP), α-smooth muscle actin (α-SMA), PDGF receptor (PDGFR), or CD90/THY1, are not always expressed at the same time (19, 20).

During cancer progression, CAFs are centrally engaged in remodeling the surrounding ECM through the secretion of ECM components such as collagens or ECM-remodeling enzymes like matrix metalloproteinases (MMPs) (18, 21, 22). This can lead to the formation of a tumor-specific ECM, which is often associated with increased tissue stiffness (23). The ECM can stimulate cancer growth and metastasis (24–26), whilst also regulating the motility and activity of tumor-infiltrating T cells (23, 27–29). Moreover, the ECM also influences the activity of other types of immune cells, including NK cells and macrophages (30–32). In addition to the CAF-mediated ECM alterations, CAFs have many other pro-tumorigenic functions, such as promotion of tumor formation, progression, and metastasis (19, 20). CAFs are involved in immunosuppression and T cell exclusion through the secretion of a variety of chemokines and cytokines, including CXCL12, CCL2, IL-6, IL-10, and TGF-β (19, 33–36) or through the upregulation of PD-L2 and FasL (37). CAFs can also secrete vascular endothelial growth factor (VEGF), which not only promotes angiogenesis, but also exerts immunomodulatory functions by inhibiting the development of dendritic cells (DCs) and downregulating their antigen-presentation abilities (38, 39).

Recently, CAFs have been shown to comprise a heterogeneous and functionally diverse cell population, with multiple CAF subsets identified (18, 20, 40). The high plasticity within the CAF population has been observed in human cancers as well as in mouse cancer models (41–43). The number of identified subsets varies between studies, but many of these describe a subset involved in ECM remodeling often termed myofibroblastic CAFs (myCAFs) and a subset with immune modulatory functions often termed inflammatory CAFs (iCAFs) (42–44).

A common tool for cancer research is the use of *in vivo* mouse tumor models. Human patient-derived xenograft (PDX) models, in which human tumor material is engrafted in mice, have become a central part of research in tumor biology and conventional cancer therapies (45). These models involve the transplantation of human cancer tissue into immune-deficient mice to avoid graft rejection. The lack of a functional immune system in these mice is not optimal for the research of immunotherapies. Genetically engineered mouse models (GEMMs) are excellent models to recapitulate the native tumor niches and provide useful insights into the interaction between malignant cells and immune effectors (46). However, GEMMs are often poorly immunogenic, and few of these models have proven to be responsive to immunotherapy (47). Syngeneic mouse tumor models, some of which were developed over 60 years ago, are economical and accessible models. The inoculated cancer cells lead to short latency periods and very fast tumor growth, which might not accurately mimic the human disease. In most cases, cells are injected subcutaneously instead of orthotopically, which could also result in differences from the human situation. Nevertheless, syngeneic tumor models have recently regained attention as reliable models for immunotherapy research since they retain a fully intact immune system and native stromal components. Consequently, syngeneic mouse models are an important approach for preclinical testing of immunotherapies, and major discoveries in the field of immunotherapy were made with the use of these mouse tumor models (48, 49).

Although developed decades ago, the immunological and molecular characteristics of syngeneic tumor mouse models and their TME have not been fully elucidated. In this work, we set out to thoroughly characterize the TME in a panel of common syngeneic mouse tumor models. Using flow cytometry and RNA sequencing (RNAseq), we characterize the tumor immune infiltrate in the different tumor models. Furthermore, we evaluate the stromal components of these models by investigating the relative tumor stiffness, collagen abundance, and the transcriptome of the CAFs. The study reveals large differences between CAFs in the different models. Comparison to established CAF subsets reveals that CAFs from the different models have similarities to specific subsets. The obtained data can contribute to improved preclinical model selection for target validation and immunotherapy drug development in future studies. Additionally, a thorough analysis of the CAFs indicates a significant contribution to the formation of an immunosuppressive TME from these cells. We show that activation of CAFs and acquisition of an immunosuppressive phenotype is driven by the tumor-infiltrating T cells.

## 2 Materials and methods

### 2.1 Cell culture of murine cancer cell lines

The murine cancer cell lines B16-F10 (melanoma), Pan02 (pancreatic ductal carcinoma), MC38 (colon carcinoma), LL2 (lung carcinoma), and CT26 (colon carcinoma) were obtained from the CCIT-DK cell biobank. EO771.LMB (breast carcinoma) was kindly gifted by Prof. Robin L. Anderson (Olivia Newton-John Cancer Research Institute, Heidelberg, Australia). The CT26 cell line is derived from a BALB/c mouse whereas the remaining cell lines are derived from C57BL/6 mice. All cell lines were tested mycoplasma-negative. Cell lines were cultured in cell culture-treated flasks (Corning, NY, USA) at 37°C and 5% CO_2_. CT26 was cultured in RPMI 1640 + GlutaMAX™, 10% fetal bovine serum (FBS), and 1% penicillin/streptomycin (P/S) (all from Gibco, Thermo Fisher, Waltham, MA, USA). B16-F10, LL2, and Pan02 were cultured in DMEM + GlutaMAX™ (Gibco, Thermo Fisher, Waltham, MA, USA), 10% FBS, and 1% P/S. EO771.LMB was cultured in DMEM, 20% FBS, 1% P/S, and 20 mM HEPES (Gibco, Thermo Fisher, Waltham, MA, USA). MC38 was cultured in DMEM + GlutaMAX™, 10% FBS, 1% P/S, 1% HEPES, 1% non-essential amino acid (NEAA) supplement, and 1% sodium pyruvate (all from Gibco, Thermo Fisher, Waltham, MA, USA). At approximately 90% confluency, the supernatant was removed, and the cells were washed twice with phosphate-buffered saline (PBS; Gibco, Thermo Fisher, Waltham, MA, USA). After washing, cells were detached by trypsinization using 0.25% Trypsin-EDTA (Gibco, Thermo Fisher, Waltham, MA, USA), and detached cells were resuspended in respective media and seeded in new culture flasks.

### 2.2 Animal experiments

Animal experiments were performed at the animal facility of the Department of Oncology, Herlev Hospital. All experiments were approved by the Danish Animal Experiment Council (license registration number 2016-15-0201-01020 and 2021-15-0201-00999). Daily care and breeding of C57BL/6 mice (C57BL/6JBomTac) and NMRI nude mice (BomTac : NMRI-*Foxn1^nu/nu^*) were performed by animal caretakers. BALB/c mice (BALB/cAnNRJ) and BALB/c nude mice (BALB/cAnNRJ-*Foxn1^nu/nu^*) were purchased from Janvier Labs (Janvier, Labs, Le Genest-Saint-Isle, France). Harvested cancer cells were counted using a hemocytometer (Hausser Scientific, Horsham, PA, USA), centrifuged at 300 g for 5 minutes at room temperature (RT) and resuspended in respective cell culture medium without supplements in the concentration of 5 x 10^5^ cells per 100 μL. Cells were placed on ice and directly before injection agitated using a pipette to ensure a homogenous cell suspension. A total of 5 x 10^5^ cells were inoculated subcutaneously using a 1 ml syringe and 25 G needle in the right flank or in both flanks of adult female mice (10-15 weeks old). Injected cells had been cultured for a maximum of 20 passages after acquisition of the cell lines. Tumor dimensions were measured three times weekly with a digital caliper, and the tumor volume was calculated using the formula volume (mm^3^) = (length)×(width)^2^/2. Mice were regularly examined for formation of ulcers on the surface of the tumors and excluded from further analysis at the presence of ulcers. The experimental endpoint was defined as tumor volume reaching 1200mm^3^. The mice were euthanized by cervical dislocation, and the tumor tissue was harvested. The excised tumors were divided into fragments and placed in digestion buffer (2.1 mg/ml collagenase type 1 (Worthington Biochemical Corporation, Lakewood, NJ, USA), 75 μg/ml DNase I (Worthington Biochemical Corporation), 5 mM CaCl_2,_ and 1% P/S in RPMI 1640 medium) for flow cytometry analysis, in RNAlater (Thermo Fisher Scientific, Waltham, MA, USA) for RNA isolation, or in 4% formaldehyde for histological staining. For shear rheology, whole tumors were placed in cold PBS and analyzed the same day.

### 2.3 Flow cytometry

Tumor fragments were placed in digestion buffer and chopped into small pieces using surgical scissors. The suspension was placed at 4°C in the dark overnight in an end-over-end rotator. The next day, the tumor digest was incubated at 37°C for 10 to 60 minutes and then homogenized by pipetting. The tumor digest was passed through a 70 μm cell strainer (Corning, NY, USA) together with PBS to obtain a single cell suspension. Erythrocytes were lysed using 2 mL red blood cell lysis buffer (Qiagen, Venlo, The Netherlands) and incubated up to 5 minutes at RT. The lysis was stopped by adding 30 mL of cell culture media. The single-cell suspension was centrifuged for 5 minutes at 300 g, the supernatant was discarded, and cells were resuspended in FACS buffer (5% bovine serum albumin (Sigma-Aldrich, St. Louis, MO), 5 mM EDTA (Sigma-Aldrich, St. Louis, MO) in PBS).

Whole tumor single-cell suspensions or purified CD45^+^ cell fractions were counted using a hemocytometer, and 5 x 10^5^ cells per sample were transferred to Falcon^®^ FACS tubes (Corning, NY, USA), resuspended in 100 μL FACS buffer containing 0.5 μL FcR blocking reagent (Miltenyi Biotec, Bergisch Gladbach, Germany), and incubated for 10 minutes at 4°C in the dark. Afterwards, samples were stained with an antibody cocktail of either the general, myeloid, or lymphoid panel (**Supplementary Table 3**). Cells were incubated with antibodies for 20 minutes at 4°C in the dark and subsequently washed with 2 mL PBS and centrifuged for 5 minutes at 300 g. A live-dead staining was included in all analyses. The supernatant was discarded, and cell suspensions were resuspended in 500 μL FACS buffer. Samples stained with the general panel underwent secondary staining with streptavidin-APC (BioLegend, San Diego, CA, USA) for 10 minutes at 4°C in the dark prior to resuspension. The samples were analyzed using the flow cytometers LSR II or FACS Canto II (BD Biosciences, San Jose, CA, USA). 50000 total events per analysis were typically recorded. For the myeloid and lymphoid panel, where a preceding CD45+ enrichment had been performed, the numbers were occasionally lower although always at least 10000 events. Time plots were inspected to ensure the flow had been stable during the analyses. Data analysis was performed with FlowJo version 10.7.1 (FlowJo LLC, Ashland, OR, USA). Gating strategies are found in **Supplementary Figures 2–4**. Fluorescence minus one (FMO) staining was performed for FAP, CD11b, CD3, CD4, CD8 and CD25. For CD206, a control staining similar to an FMO was performed but with the inclusion of an isotype control antibody conjugated with the same fluorophore as the anti-CD206 antibody. To avoid spillover of emission signals from other channels, all panels underwent a compensation procedure prior to running samples, using the BD FACS Diva software version 8.0.1 (BD Biosciences, San Jose, CA, USA). A list of antibodies can be found in **Supplementary Table 3**.

### 2.4 Purification of CD45^+^ cells

To enrich the leukocyte population of whole tumor suspensions for flow cytometry analysis or FACS sorting, the CD45^+^ population was enriched by magnetic-activated cell sorting. Single-cell suspensions were counted, centrifuged at 300 g for 5 minutes at RT, and then resuspended in 90 μL of FACS buffer with 10 μL FcR blocking reagent (Miltenyi Biotec) per 10^7^ cells. Cells were incubated for 10 minutes at 4°C and subsequently labeled with 10 μL CD45 MicroBeads (Miltenyi Biotec, Bergisch Gladbach, Germany) per 10^7^ cells and incubated for 20 minutes at 4°C. After incubation, cells were washed once with FACS buffer and loaded onto LS columns (Miltenyi Biotec, Bergisch Gladbach, Germany) placed in a magnetic MACS MultiStand (Miltenyi Biotec, Bergisch Gladbach, Germany). The column was washed once with 2 mL FACS buffer, and subsequently the CD45^+^ fraction was eluted from the column.

### 2.5 Fluorescence activated cell sorting

The CD45^-^ fraction left after CD45^+^purification was used to sort FAP^+^ CAFs from the tumors. CD45^-^ cell suspensions were stained with a live/dead marker and FAP-Biotin (R&D Systems, Minneapolis, MN, USA) followed by secondary staining with streptavidin as described above. Up to 3 x 10^5^ cells were sorted into PBS. The samples were sorted using a FACS Aria I cell sorter (BD Biosciences, San Jose, CA, USA). Immediately after sorting, cells were processed for RNA isolation.

### 2.6 Shear rheology

The relative stiffness of tumors was measured by shear rheology using a DHR-2 rotational rheometer (TA Instruments, New Castle, DE, USA). After excision, tumors were kept in PBS on ice and measurements were taken the same day. Tumors were cut using a scalpel, and disks of 8-mm diameter were obtained using a biopsy punch. Measurements were performed using an 8-mm parallel plate geometry at 21°C, at a fixed angular frequency of 1 rad/s, and an increasing strain from 0.1 to 2%. Storage modulus (G′) was extracted at 0.3% strain (shown to be within the linear viscoelastic response range).

### 2.7 RNA isolation

RNA was isolated from sorted cells or tumor fragments using the RNeasy^®^ Mini Kit (Qiagen, Venlo, The Netherlands) according to the manufacturer’s protocol. When isolating RNA from sorted cells, the cells were centrifuged for 5 minutes at 300 g and lysed by resuspension in 350 μL RLT lysis buffer. When isolating RNA from tumor tissue, a tumor fragment of approximately 30 mg was placed in a 2 mL microcentrifuge tube with 500 µL RLT buffer containing 1:100 β-mercaptoethanol and a 5 mm stainless steel bead. The tumor fragment was homogenized with a TissueLyser (Qiagen, Venlo, The Netherlands) for 3 minutes at a frequency of 25/s. The bead was removed, and the samples were spun down at top speed (21,000 *g*) for 3 minutes. A volume of 350 µL lysate was used for RNA isolation. Isolated RNA was stored at -80°C until further analysis. Concentration, purity, and integrity of RNA extracts were measured using the Agilent RNA 6000 Nano Kit and the Agilent 2100 Bioanalyzer (both from Agilent Technologies, Santa Clara, CA, USA) according to manufacturer’s instructions.

### 2.8 qRT-PCR

Reverse transcription of RNA was done using the iScript cDNA Synthesis Kit (Bio-Rad Laboratories, Hercules, CA, USA) according to the manufacturer’s instructions to obtain complementary DNA (cDNA). Controls without reverse transcriptase and controls without template were included. The quantitative real-time PCR (qRT-PCR) was done using the Brilliant III Ultra-Fast SYBR Green QPCR Master Mix (Agilent Technologies, Santa Clara, CA, USA) according to the manufacturer’s instructions. The program used was: 3 minutes at 95°C, 40 cycles of 5 seconds at 95°C, 40 cycles of 20 seconds at 60°C, and a melting curve analysis of 65-95°C with 0.5°C increment, 5 seconds per step. qRT-PCR was performed using an AriaMX Real-Time PCR System (G8830A, Agilent Technologies, Santa Clara, CA, USA). Samples were run in triplicates and normalized to the internal reference gene, *Actb*. Relative fold changes were calculated using the comparative cycle threshold (ΔΔCT) method. Primers were designed using the Primer-BLAST tool ((National Center for Biotechnology Information, National Institutes of Health, Bethesda, MD, USA). Primer sequences are listed in **Supplementary Table 4**.

### 2.9 RNA sequencing and analysis

A total of 1000 ng RNA from tumor fragments and 400 ng RNA from sorted CAFs was prepared for sequencing using polydT enrichment according to the manufacturer’s protocol (Illumina, San Diego, CA, USA). Library preparation was performed using the NEBNext RNA library prep kit (Illumina, San Diego, CA, USA). The library quality was assessed using a Fragment Analyzer (Agilent, Santa Clara, CA, USA) followed by library quantification using the Illumina library quantification kit. Sequencing was done on a NovaSeq 6000 platform (Illumina, San Diego, CA, USA). Sequenced reads were aligned to the mouse reference mm10 genome using STAR, version 2.5.0 (50). The gene expression count matrix was generated using HOMER (51).

All analyses were performed with R. Differential expression analysis was performed using the DESeq2 package. Principal component analysis was performed using the prcomp package. z-score normalized RPKM values of selected genes were used to generate heatmaps using the pheatmap package. Fuzzy clustering was performed using VSClust (52). The gene lists used for illustrating myeloid factors, lymphoid factors, and stromal factors were based on gene lists by NanoString Technologies. The gene lists used for illustrating collagens and core matrisome genes were from the matrisomeDB (53, 54). The gene lists used for illustrating immunosuppressive factors and MMPs were self-generated.

### 2.10 CIBERSORT analysis

The computational framework of the CIBERSORT analytical tool (55), along with the developed ImmuCC signature matrix (non-tissue specific) (56), suitable for the deconvolution of mouse bulk RNA-Seq data, were used to characterize and quantify 25 immune cell subtypes. The ImmuCC signature matrix consists of 511 genes and 489 genes from our bulk RNAseq data were mapped (22 missing).

For deconvolution of the bulk RNAseq samples with CIBERSORT, RPKM pre-normalized data were used to produce the input mixture matrix. Additionally, the analysis included both CIBERSORT-Relative and CIBERSORT-Absolute modes. While CIBERSORT-Relative represents immune cell fractions, which are relative to the total immune content, therefore suitable for intra-sample comparisons, CIBERSORT-Absolute produces a score that quantifies the abundance of each cell type, making it appropriate for intra-sample comparisons between cell types as well as inter-sample comparisons of the same cell type. The CIBERSORT outputs were generated by performing 1000 permutations and by disabling the quantile normalization parameter.

For the purposes of this study, three population schemes were defined, resulting in the aggregation of some of the 25 immune sub-populations. Furthermore, the CIBERSORT estimates were averaged across cell line replicates, to generate one estimate/score per cell line.

Total absolute scores for sub-populations merged were calculated as the sum of the sub-populations. The relative fractions were re-calculated based on the new total immune content of each scheme. The CIBERSORT software source code in R was obtained from the website: https://cibersort.stanford.edu/, after registration and request for access and download.

### 2.11 Histology

Tumors were fixed in 4% formaldehyde overnight at 4°C. Samples were transferred to 70% ethanol and stored at 4°C until paraffin embedding. Tissues were embedded in paraffin and cut into 3.5 μm tissue sections. Sections were deparaffinized with xylene and hydrated through ethanol/water dilutions. For the detection of fibrillar collagen, sections were stained with 0.1% Sirius red diluted in saturated picric acid (Ampliqon, Odense, Denmark) and counterstained with Weigert’s hematoxylin. Images of stained sections were acquired using a light microscope with polarization filters. Picrosirius red (PSR) positive areas were quantified with Qupath software (ver. 0.2.3) (57) using the Qupath Pixel Classifier with full resolution. Positive and negative areas were manually assigned on several sections, and these were used to train the Pixel Classifier until it could reliably detect positive areas. Ten squares were randomly distributed across each section and the trained Pixel Classifier was run on these. The average of the 10 squares was determined for each section.

### 2.12 Statistics

Data analyses, statistical analyses, and graph generations were performed with Prism 8 (GraphPad) unless otherwise stated. Correlations between percentage collagen positive area and storage modulus and percentage FAP^+^ CAFs were assessed by Pearson rank correlations. Asterisks in the graphs indicate significance as described in respective figure legends. Differences were considered statistically significant at p < 0.05.

## 3 Results

### 3.1 Murine tumor models display distinct gene expression profiles indicative of differences in the tumor microenvironment

**Figure 1:**
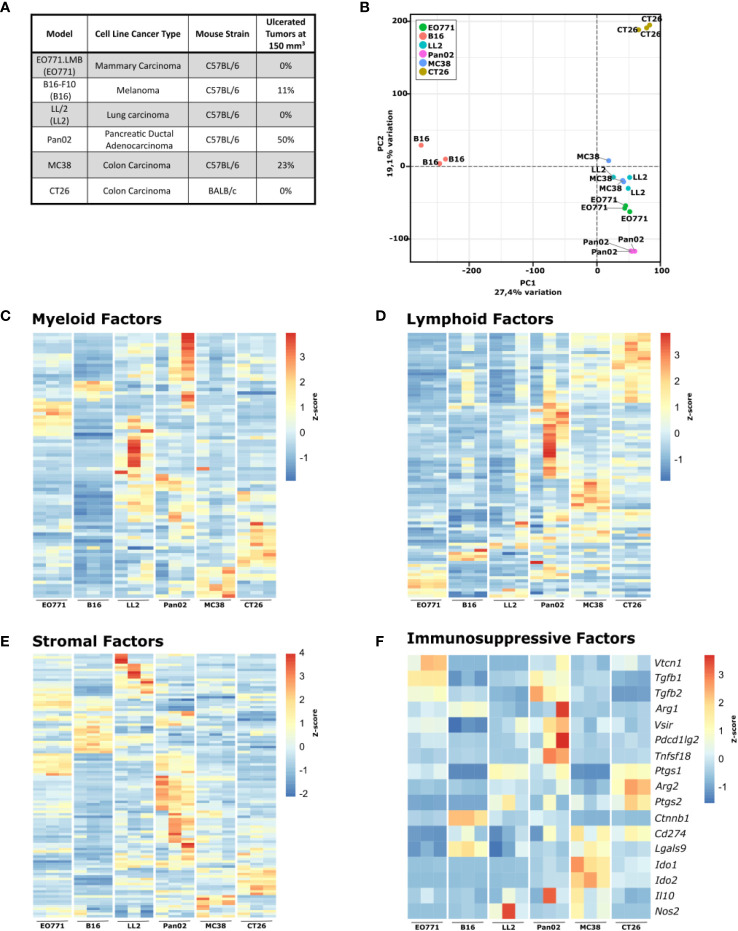
Six commonly used murine syngeneic tumor models show distinct gene expression profiles. **(A)** Table summarizing the tumor models, cancer type, origin, and rate of ulceration. **(B)** Principal component analysis (PCA) plot of RNA isolated from tumor fragments derived from the indicated cell lines. C-F) Heatmaps of normalized (Z-score) RNAseq read counts of genes encoding myeloid factors **(C)**, lymphoid factors **(D)**, stromal factors **(E)**, and immunorsuppressive factors **(F)**.

To study the TME formed in different mouse tumor models, we selected a panel of commonly used syngeneic mouse models representative of some of the most common human cancer types (**Figure 1A**). Five of the murine cell lines (EO771.LMB, B16-F10, LL/2, Pan02, and MC38) are derived from C57BL/6 mice, while CT26 is derived from BALB/c mice (**Figure 1A**). B16-F10 will be referred to hereafter as B16, EO771.LMB as EO771, and LL/2 as LL2. The inoculated B16, LL2, MC38 and CT26 cells all formed fast growing tumors, whereas EO771 and Pan02 displayed slower growth kinetics (not shown). Pan02 tumors grew particularly slowly and had a strong tendency to develop severe tumor ulceration. When tumor volumes exceeded 150 mm^3^, 50% of mice with Pan02 tumors had developed ulcers compared to maximum 23% of other tumor-bearing mice (**Figure 1A**). Ulcerating tumors were excluded from subsequent analyses of the TME.

For a detailed comparison of the six tumor models, we initially analyzed the tumors by RNAseq (full gene expression dataset in **Supplementary Table 1**). A principal component analysis (PCA) highlights the profound differences in the gene expression pattern between the tumor models (**Figure 1B**). The transcriptomic differences were expected since the used cancer cell lines originate from different tissues, but analysis of the expression of genes related to myeloid cells (**Figure 1C**) and lymphoid cells (**Figure 1D**) indicated that formation of distinct TMEs also contributed to the observed global gene expression differences. All tumor models have discrete expression profiles of genes related to both myeloid and lymphoid cells, with B16 and EO771 tumors having gene expression profiles indicative of low immune infiltration. Expression analysis of genes encoding stromal factors also showed large differences in the stromal compartment between the tumor models (**Figure 1E**). To investigate the influence of tumor-infiltrating T cells on tumor growth, B16, Pan02, MC38, and CT26 cells were subcutaneously injected into athymic nude mice that lack functional T cells and at the same time into immunocompetent wildtype mice. MC38 and CT26 cancer cells harbor many mutations and are considered immunogenic tumor models (58). As expected, MC38 and CT26 grew substantially faster in nude mice, suggesting high immunogenicity of these tumor models (**Supplementary Figures 1A, B**). B16 and Pan02 tumor growth were not different in nude mice compared to wildtype mice (**Supplementary Figures 1C, D**). Interestingly, gene expression analysis of a panel of immunosuppressive factors showed obvious differences between the tumor models, indicating that they depend on different mechanisms for immune escape (**Figure 1F**). Even between the two immunogenic colorectal cancer models MC38 and CT26, clear differences were observed. MC38 tumors have a particularly high expression of *Ido1* and *Ido2*, whereas CT26 tumors express high levels of *Arg2*, *Ptgs1*, and *Ptgs2* encoding arginase-2, COX-1, and COX-2, respectively.

### 3.2 Flow cytometry analysis of the TME unveil different immune cell compositions between tumor models

To further characterize the distinct TME of the six tumor models, we analyzed the cellular composition of single cell suspensions of tumors by flow cytometry. The TME was characterized with a special focus on immune cell populations but also on the presence of other cell types such as CAFs and endothelial cells (for gating strategy see **Supplementary Figures 2–4**).

**Figure 2:**
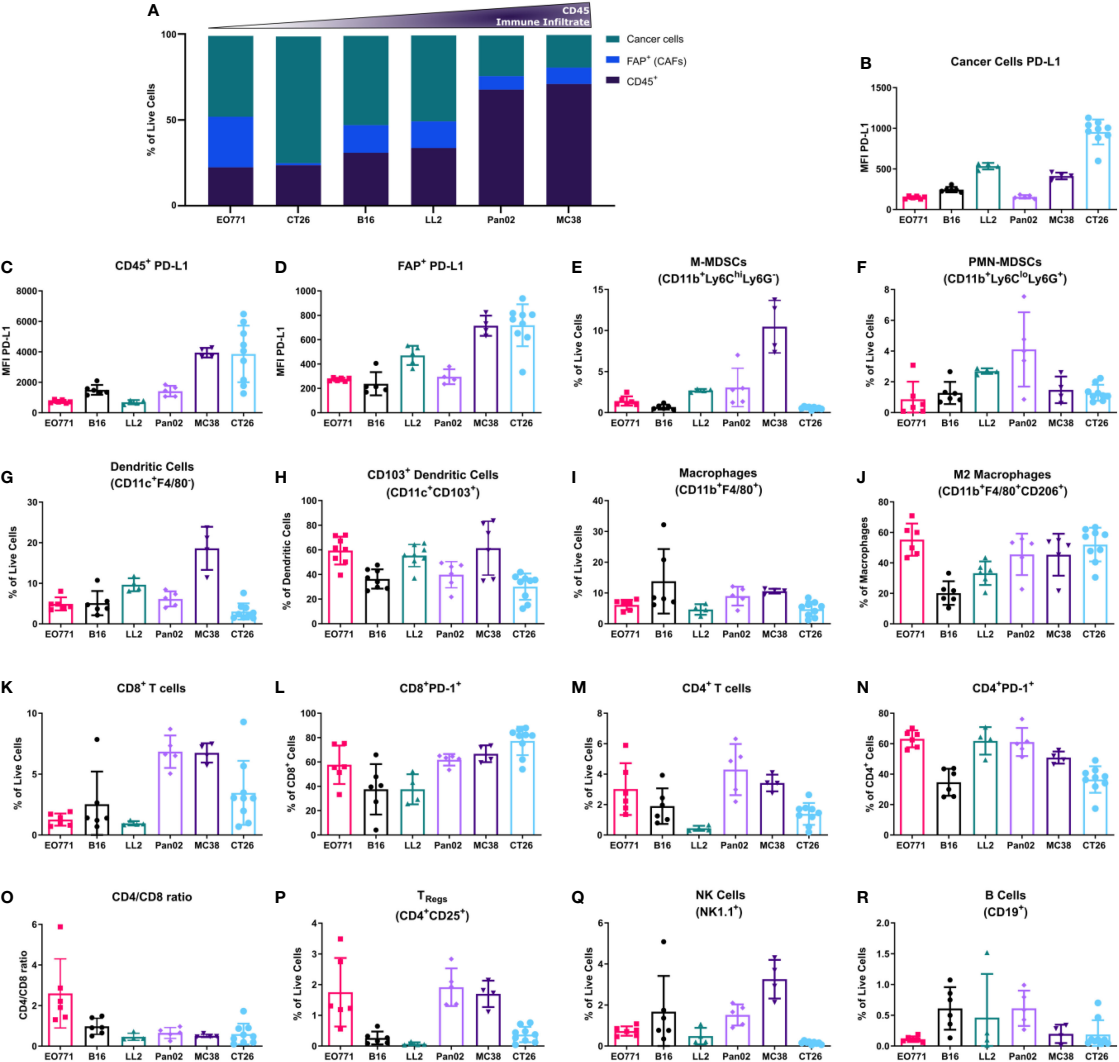
Flow cytometry analysis of the tumor microenvironment unveils different composition of immune populations across tumor models. **(A)** Histogram summarizing the median abundance (% of live cells) of cancer cells, CAFs, and immune cells; (B-R) PD-L1 median fluorescence intensity (MFI) expression on cancer cells **(B)**, CD45+ cells **(C)**, and FAP+ CAFs **(D)**; percentage of M-MDSCs **(E)**, PMN-MDSCs **(F)**, and dendritic cells **(G)** out of living cells; percentage of CD103+ dendritic cells out of all dendritic cells **(H)**; percentage of macrophages out of living cells **(I)**; percentage of M2-like macrophages out of all macrophages **(J)**; percentage of CD8+ T cells out of living cells **(K)**; percentage of PD-1+ CD8+ T cells out of all CD8+ T cells **(L)**; percentage of CD4+ T cells out of living cells **(M)**; percentage of PD-1+ CD4+ T cells out of all CD4+ T cells **(N)**; CD4/CD8 ratio **(O)**; percentage of TRegs **(P)**, NK cells **(Q)**, and B cells **(R)** out of living cells. **(A-D)** are based on the general flow cytometry panel, **(E-J)** are based on the myeloid flow cytometry panel, and **(K-R)** are based on the lymphoid flow cytometry panel.

First, the overall immune infiltrate defined as CD45^+^ cells was quantified as well as non-immune cells such as CAFs (**Figure 2A**). The tumor models showed large variations in CD45^+^ cell infiltration, extending from poorly immune-infiltrated tumor models (EO771, CT26, and B16) to highly immune-infiltrated models such as MC38 and Pan02 (**Figure 2A**). The percentage of CAFs varied greatly between tumor models with the breast cancer model EO771 displaying the highest amount of CAFs (**Figure 2A**). Upregulation of PD-L1 by cancer cells or myeloid cells including tumor-associated macrophages (TAMs) is a common mechanism employed by tumors for evading immune-mediated elimination (2). Our analysis showed that PD-L1 expression was generally lower on cancer cells (CD45^-^FAP^-^CD31^-^) compared to immune cells (CD45^+^) (**Figures 2B, C**), but with some tumor models such as CT26 having a relatively high level of PD-L1 expression on cancer cells (**Figure 2B**). The expression of PD-L1 on immune cells followed a similar pattern with highest expression in MC38 and CT26 tumors (**Figure 2C**). Moreover, we observed expression of PD-L1 by CAFs, with a particularly high expression of PD-L1 on CAFs from CT26 and MC38 tumors (**Figure 2D**).

To expand our characterization of the TME, we analyzed the composition of tumor-infiltrating immune cells. Myeloid cells were divided into monocytic myeloid-derived suppressor cells (M-MDSCs) (CD11b^+^F4/80^-^Ly6C^hi^Ly6G^-^), polymorphonuclear (PMN)-MDSCs (CD11b^+^F4/80^-^Ly6C^lo^Ly6G^+^), dendritic cells (F4/80^-^CD11c^+^), and TAMs (CD11b^+^F4/80^+^). The M-MDSCs comprised a large fraction of the total number of cells in MC38 tumors and a low fraction in B16 and CT26 tumors (**Figure 2E**). In contrast, PMN-MDSCs made up a small fraction of cells in MC38 tumors and instead a larger fraction in the Pan02 and LL2 tumor model (**Figure 2F**). Dendritic cells made up a large fraction of cells in LL2 and MC38 tumors (**Figure 2G**). CD103^+^ dendritic cells are critical for the generation of anti-tumor immune responses (59). We therefore analyzed the fraction of dendritic cells that belonged to this subset across the tumor models. In EO771, LL2, and MC38 tumors, CD103+ dendritic cells comprised a large fraction of dendritic cells, whereas this fraction was slightly smaller in the B16, Pan02, and CT26 tumors (**Figure 2H**).

TAMs made up a comparable proportion of cells across most of the tumor models, with a slightly larger fraction in B16 and MC38 tumors (**Figure 2I**). The fraction of TAMs that were CD206^+^, which is an indicator of M2 polarization, was smallest in B16 tumors followed by LL2 tumors and equally large in the other models (**Figure 2J**).

The infiltration of lymphoid cells in the TME was also assessed. Notably, the largest amount of CD8^+^ T cells was observed in Pan02, MC38, and CT26 tumors (**Figure 2K**). Within the CD8^+^ T cell population, the proportion of PD-1^+^ cells was highest in CT26 tumors and lowest in B16 and LL2 tumors (**Figure 2L**). CD4^+^ T cells were most abundant in EO771, Pan02, and MC38 tumors (**Figure 2M**). The fraction of PD-1^+^CD4^+^ T cells was largest in EO771, LL2, and Pan02 tumors (**Figure 2N**). The differences in infiltration of CD8^+^ and CD4^+^ T cells led to a CD4/CD8 ratio that was high in EO771 tumors, intermediate in B16 tumors, and low in the other tumor models (**Figure 2O**). The highest numbers of CD25^+^CD4^+^ T cells, which in mice primarily represent regulatory T cells (T_regs_) (60), were observed in EO771, Pan02, and MC38 tumors (**Figure 2P**). NK cells were most abundant in B16, Pan02, and MC38 tumors and almost completely absent in CT26 tumors (**Figure 2Q**). The number of tumor-infiltrating B cells was very low in all models although slightly higher in B16, LL2, and Pan02 tumors (**Figure 2R**).

### 3.3 Transcriptomic analyses uncover clear immunological diversity between tumor models

To further analyze the immune composition of the TME, we took advantage of the ability to estimate the relative abundance of different cellular components based on RNAseq data. Using the CIBERSORT tool (55), which allows for quantifications of cell fractions from bulk tissue gene expression profiles, we first compared the cell type abundances obtained from RNAseq and flow cytometry.

**Figure 3:**
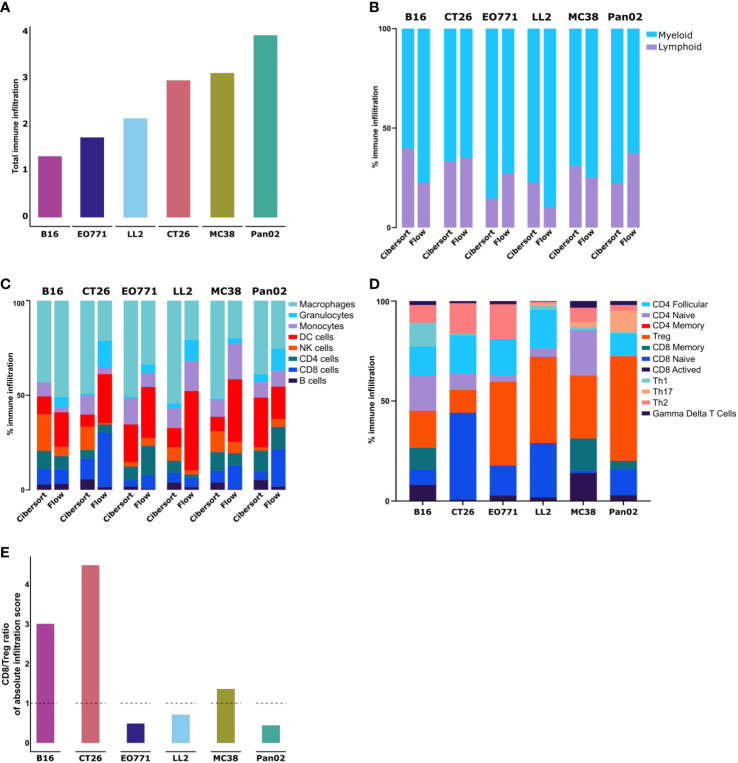
Transcriptomic analysis of whole tumors shows differences in immune cell composition between models. **(A)** Absolute amount of immune infiltration across six tumor models based on RNA isolated from tumor fragments analyzed using the CIBERSORT tool. **(B)** Ratio of lymphoid to myeloid cells based on RNAseq data analyzed using the CIBERSORT tool. **(C)** Comparison of immune cell population abundancies estimated from CIBERSORT and flow cytometry. **(D)** Abundancies of specific T cell subsets across the tumor models. **(E)** Ratio of CD8 to T^regs^ based on absolute infiltration score.

The absolute amount of immune infiltration in the tumors estimated using CIBERSORT was largely in line with the CD45^+^ quantification by flow cytometry with the only exception of CT26 that based on the RNAseq data appeared more immune infiltrated compared to the flow cytometry-based analysis (**Figure 3A**, compare to **Figure 2A**). The estimated ratio of lymphoid to myeloid cells was also comparable to the flow cytometry data (**Figure 3B**). Additionally, we observed that the composition of the myeloid compartment differed quite a lot with for instance macrophage infiltration estimated substantially higher by CIBERSORT compared to the quantification by flow cytometry in all the models, except for the B16 tumors (**Figure 3C**). The composition of the lymphoid compartment was similar, although the number of NK cells was generally estimated to be higher compared to our flow cytometry data (**Figure 3C**).

Next, we utilized CIBERSORT to analyze T cell subsets. Notable differences in the composition of T cell subsets were observed between the tumor models (**Figure 3D**). The highest fractions of activated CD8^+^ T cells were found in B16 and MC38 tumors, while CT26 tumors had the largest fraction of naïve CD8^+^ cells (**Figure 3D**). Across all models, CT26 tumors contained the lowest fraction of T_regs_, while this population accounted for larger proportions in Pan02, LL2, and EO771 tumors (**Figure 3D**). The predicted ratio of CD8^+^ T cells to T_regs_ was particularly high in CT26 and B16 tumors (**Figure 3E**).

### 3.4 Collagen content and tissue stiffness vary profoundly between tumor models

The ECM constitutes an important component of the TME with multiple tumor-promoting properties (61), including the suppression of immune-mediated killing of cancer cells (23, 27–30). The quantitatively dominant ECM component is collagen type I, which is also an important contributor to increased tissue stiffness of solid tumors.

**Figure 4:**
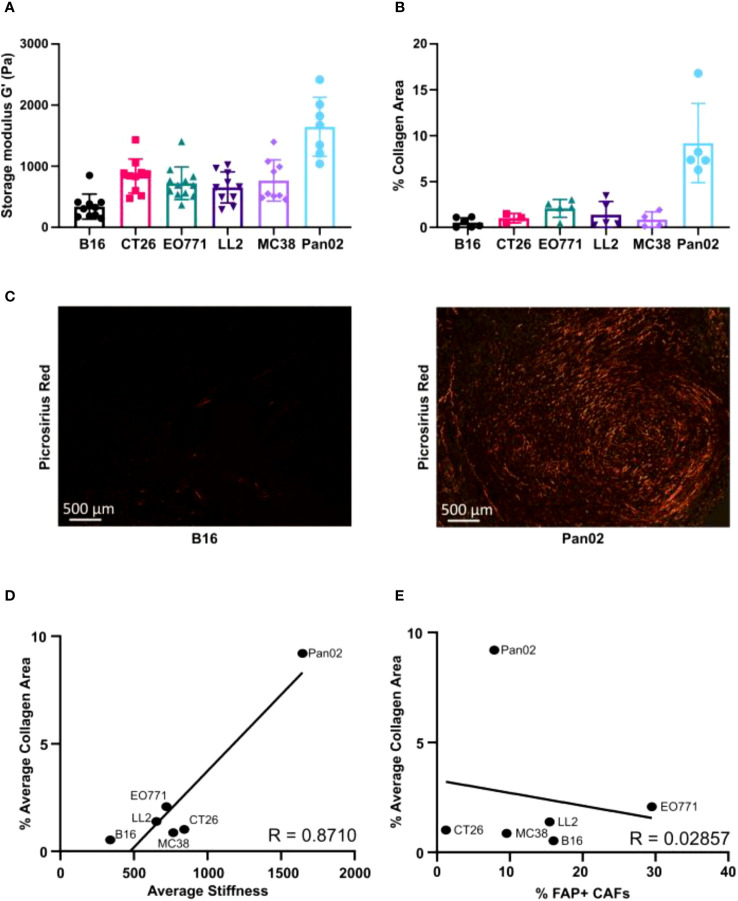
Relative extracellular matrix stiffness measurements by shear rheology varies profoundly between models. **(A)** Measurements of mechanical stiffness (storage modulus) for all the tumor models (n = 7-12). **(B)** Quantification of collagen based on picrosirius red (PSR) staining of paraffin-embedded tissue sections (n = 3-6). **(C)** Representative images of PSR staining in B16 (left) and Pan02 (right) tumor sections. **(D)** Correlation between storage modules (stiffness) and percentage of collagen positive area, analyzed by Pearson rank correlation (R = 0.8710). **(E)** Correlation between percentage of FAP+ CAFs and percentage of collagen positive area analyzed by Pearson rank correlation (R = 0.02857).

The mechanical stiffness of excised tumors was measured using shear rheology. Pan02 tumors had the highest tissue stiffness, while B16 tumors had the lowest stiffness (**Figure 4A**). All the other models displayed intermediate levels of stiffness (**Figure 4A**). Picrosirius red (PSR) staining was used to visualize and quantify fibrillar collagen-positive areas in paraffin-embedded tissue sections (**Figures 4B, C**). Pan02 tumors also had the highest levels of intratumorally deposited fibrillar collagen, and B16 tumors had the lowest levels (**Figures 4B, C**). In alignment with these quantifications, a positive correlation between the collagen-positive area and tumor stiffness was observed (**Figure 4D**). CAFs are the main producers of collagen type I in the TME, but the collagen-positive area did not correlate with the number of tumor-infiltrating FAP^+^ CAFs (**Figure 4E**). This suggests that CAFs in the different models can acquire distinct phenotypes, which contribute to collagen deposition and tumor stiffness to various degrees.

### 3.5 Cancer-associated fibroblasts display model-specific transcriptional programs

**Figure 5:**
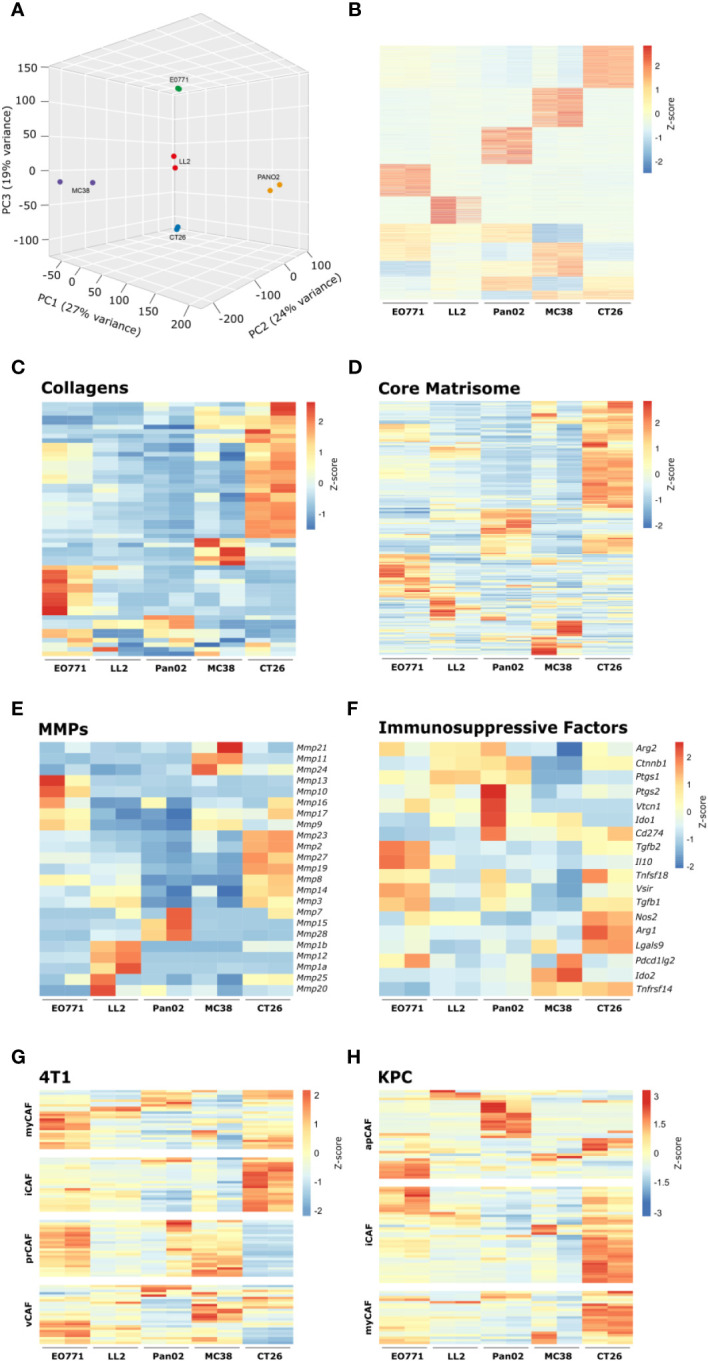
Transcriptomic analysis of isolated CAFs reveal model-specific transcriptional programs. **(A)** 3D PCA analysis based on RNAseq of FACS-isolated CAFs from tumors from five tumor models. **(B)** Fuzzy clustering analysis of RNA from isolated CAFs. **(C-F)** Heatmaps of normalized (Z-score) RNAseq read counts of genes encoding collagens **(C)**, core matrisome proteins **(D)**, MMPs **(E)**, and immunosuppressive factors **(F)**. **(G, H)** Comparison of RNAseq data from isolated CAFs from the indicated models with previously described CAF subsets from murine 4T1 breast cancer42 **(G)** and murine pancreatic cancer41 **(H)**.

The cell-surface serine protease FAP is a commonly used CAF-marker that is expressed on the majority of identified CAF subsets (43). To examine if CAFs acquire distinct phenotypes in the different tumor models, FAP^+^ CAFs were FACS-sorted for whole transcriptome analyses. For each tumor model, RNA was successfully obtained from two individual rounds of cell sorting, with the exception of B16 tumors from which we were unable to obtain RNA of sufficiently high quality. The gene expression dataset can be found in **Supplementary Table 2**. Based on a scree plot (**Supplementary Figure 5**), it was apparent that 3 principal components were describing most of the variation between samples. Therefore, we created a 3D PCA plot, which showed that the transcriptional program of CAFs varies between the different tumor models (**Figure 5A**). Fuzzy clustering analysis confirmed that the CAFs have distinct tumor model-specific gene expression profiles (**Figure 5B**). CAFs are centrally engaged in ECM remodeling associated with cancer progression (19). To investigate the tumor model-specific role of CAFs in these processes, the gene expression profiles of collagens and core matrisome genes were examined (**Figures 5C, D**). The analysis clearly showed that CAFs contribute to ECM production in solid tumors in unique ways depending on the tumor model. Moreover, the gene expression profile of genes encoding MMPs, which are critical enzymes for the cancer-associated degradation of ECM components, differed dramatically between tumor models (**Figure 5E**). In addition to the well-established role of CAFs in ECM remodeling, a growing body of evidence indicates that CAFs can also acquire an immunosuppressive phenotype and thereby promote tumor growth. Interestingly, a comparison of the expression of genes known to be involved in immunosuppression in the TME showed that CAFs had very different patterns of expression of these genes depending on the tumor model (**Figure 5F**). In the CT26 tumor model, CAFs expressed high levels of a range of transcripts encoding immunosuppressive molecules, including *Cd274*, *Tgfb1*, *Nos2*, *Arg1*, and *Lgals9*. This indicates that these CAFs could be important for inhibiting T cell activity in the TME.

To investigate how CAFs from the individual tumor models relate to CAF subsets described by others, we compared our RNAseq data to gene signatures based on single cell sequencing of murine 4T1 breast cancer (43) or murine KPC pancreatic cancer (42). The comparison did not show a perfect overlap between CAFs from the tumor models and the specific subsets but nevertheless indicated a tumor model-dependent skewing toward certain subsets (**Figures 5G, H**). CAFs from MC38 tumors showed similarities with vascular CAFs (vCAFs) and proliferating CAFs (prCAFs) identified in murine 4T1 breast tumors (**Figure 5G**). CAFs from Pan02 tumors showed similarities with antigen-presenting CAFs (apCAFs) identified in KPC tumors (**Figure 5H**). CAFs from CT26 tumors appeared very similar to the myofibroblastic CAFs (myCAFs) and inflammatory CAFs (iCAFs) identified in both 4T1 and KPC tumors (**Figures 5G, H**).

### 3.6 Tumor-infiltrating T cells promote an immunosuppressive CAF phenotype

**Figure 6:**
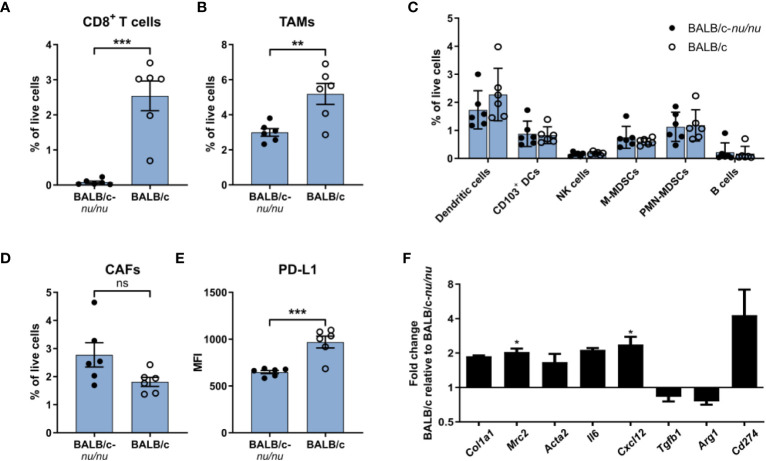
The immunosuppressive phenotype of CAFs is induced by tumor-infiltrating T cells. Flow cytometry analysis of CT26 tumors from athymic BALB/c nude (BALB/c nu/nu) mice (filled circles) and BALB/c mice (empty circles). **(A)** Percentage of CD8+ T cells out of live cells. **(B)** Percentage of TAMs (CD11b+F4/80+) out of live cells. **(C)** Percentage of six immune cell populations out of live cells. **(D)** Percentage of CAFs (CD45-FAP+) out of live cells. **(E)** PD-L1 MFI expression of CAFs. n = 6. **(F)** qRT-PCR analysis of a panel of genes associated with activation or immunosuppression in sorted FAP+ CAFs. Error bars indicate SEM. Statistical analysis was performed by two-tailed Student’s t-test. *** = p ≤ 0.001, ** = p ≤ 0.01, * = p ≤ 0.05, not significant when p > 0.05. In review

CAFs from CT26 tumors had a distinct gene expression profile indicative of high immunosuppressive activity (**Figures 5F–H**) and expressed higher levels of cell surface PD-L1 (**Figure 2B**). To investigate the importance of T cell infiltration for acquisition of the specific CAF phenotype in CT26 tumors, we compared tumors established in immunocompetent BALB/c mice and in T cell-deficient athymic BALB/c nude mice. Flow cytometry analysis of the TME confirmed that CD8^+^ T cells were absent in the nude mice (**Figure 6A**). The absence of CD8^+^ T cells was accompanied by a reduction in TAM infiltration (**Figure 6B**), whereas the abundance of other cell populations was unaffected (**Figure 6C**). The number of CAFs was similar between tumors from immunocompetent and nude mice (**Figure 6D**), but PD-L1 expression on CAFs was lower in the absence of CD8^+^ T cells (**Figure 6E**).

To further characterize the CAFs, FAP^+^ cells were FACS-isolated from CT26 tumors from immunocompetent mice and from nude mice (**Supplementary Figure 6**) and analyzed by qRT-PCR. There was a trend towards upregulation of genes encoding fibroblast activation markers (*Col1a1, Mrc2, Acta2*) in the presence of tumor-infiltrating CD8^+^ T cells (**Figure 6F**). We also assessed the expression of five genes implicated in CAF-mediated immune suppression. Among these, *Cxcl12* was significantly upregulated in the immunocompetent tumors, and there was a clear trend towards an upregulation of *Il6* and *Cd274* (PD-L1) (**Figure 6F**). These results suggest that tumor-infiltrating T cells could be critically involved in promoting activation of CAFs as well as an immunosuppressive CAF phenotype in the immunogenic CT26 tumor model.

## 4 Discussion

In this study, we compared the TMEs of six commonly used syngeneic mouse tumor models. For optimal comparison of the tumor models we exclusively used female mice, which are also easier than males to keep in harmonious groups. We cannot exclude that gender-dependent differences between the models could exist. Although several of the models have comparably fast tumor growth kinetics and appear macroscopically similar, we observed striking differences in the formed TME. RNAseq of dissected tumor tissue showed distinct gene expression profiles of each of the six tumor models. This information can potentially be utilized for the rational selection of appropriate tumor models for future studies. The distinct gene expression profiles could in part be due to the diversity in the cancer cell lines’ tissue origins. However, further analysis of genes related to myeloid, lymphoid, and stromal cells indicated that distinct cellular composition of the TMEs also contributed to the observed differences. We also identified large differences in the expression of immunosuppressive genes, suggesting the acquisition of tumor model-specific mechanisms of immune escape. For instance, *Ido1* and *Ido2* are highly expressed in MC38 tumors, whereas Pan02 tumors have a strong TGF-β signature. Among the tumor models in our panel, MC38 and CT26 tumors, which are the most immunogenic, displayed the highest levels of PD-L1 gene expression.

Analysis of the cell composition of the TME using flow cytometry confirmed that the tumor models were infiltrated with highly varying numbers of immune and stromal cells. Even the two colon cancer models, CT26 and MC38, were dramatically different, with MC38 tumors being much more immune infiltrated than CT26 tumors. The MC38 and CT26 tumor models are commonly used for immunotherapy research and knowledge about the distinct TMEs of these two models should be taken into account since different strategies could be required for successful immunotherapeutic efficacy in the two colorectal tumor models. It should be noted that the different strains of mice used for the MC38 (C57BL/6) and CT26 tumors (BALB/c) could contribute to some of the observed differences. In addition to the overall level of immune infiltration, large differences in the composition of immune cells in the individual tumor models were observed. For instance, MC38 tumors contained high numbers of M-MDSCs whereas PMN-MDSCs were most abundant in LL2 and Pan02 tumors. It should be noted that we quantified PMN-MDSCs and M-MDSCs based solely on surface markers that would also identify granulocytes and monocytes, respectively (62). Further analyses would be needed to characterize and confirm the immunosuppressive activity of these cells.

As an alternative to flow cytometry-based profiling of the tumors, we also estimated the immune composition from bulk RNAseq data using the CIBERSORT tool. In regard to the overall infiltration of myeloid and lymphoid cells, the results were well in line with the flow cytometry-based profiling. However, the RNAseq-based estimation of the relative abundance of different myeloid cell types showed some discrepancies with the flow cytometry analysis. Although the flow cytometry analyses were limited by a relatively low number of surface markers to distinguish the individual cell populations, we speculate that a main reason for the discrepancy between the flow cytometry- and RNAseq-based analyses is that the gene signatures used to estimate cell type abundancies still require refinement. The tool is, however, extremely valuable in the absence of the possibility of flow cytometry analysis. In this study, the CIBERSORT tool enabled us to estimate the abundance of lymphocyte subsets for which required markers were not included in the flow cytometry analysis.

Our characterization of the TME complements previous reports investigating the immune cell composition in various tumor models based on flow cytometry or RNA sequencing (58, 63–65). Although some variations between the different studies are seen, our data is largely in line with these reports. Collectively, these studies form an excellent framework for the rational selection of appropriate tumor models for future cancer immunotherapy research. In addition to a characterization of the immune cell composition in tumors, we have in our study also examined the number and phenotype of infiltrating CAFs as well as the tissue stiffness of the tumor models. These are components of the TME that can influence invasive tumor growth directly and modulate immune activity in the tumors.

An increased matrix stiffness, which is also reflected in increased tissue stiffness, has been strongly associated with aggressive tumor growth and poor prognosis in patients with gastrointestinal and breast cancer (66–69). Although the pro-tumorigenic role of increased matrix stiffness has been studied extensively, surprisingly little is known about the relative tissue stiffness of common syngeneic tumor models. Here, we showed that Pan02 tumors had the highest fibrillar collagen content and tissue stiffness, whereas B16 tumors had very low collagen content and tissue stiffness. The collagen content did not correlate with the number of CAFs, and indeed the CAFs in the different tumor models also displayed very distinct phenotypes. CAFs appeared to differ dramatically in their ECM remodeling abilities based on gene expression profiles.

Whole-transcriptome analysis of CAFs from the characterized tumor models suggested that they can acquire very different immunosuppressive functions. A comparison to CAF subset gene signatures obtained from studies of murine breast and pancreatic cancer showed some similarities to these subsets depending on the tumor model. The model-dependent skewing towards specific CAF subsets did, however, not give any further insight into the different collagen-levels observed in the tumor models. CAFs from CT26 tumors showed resemblance to myCAFs and iCAFs, which was in accordance with the high expression of matrisome genes and genes involved in immunosuppression, including *Cd274* encoding PD-L1. It is still not well understood how large a role PD-L1 expressed by CAFs plays for the suppression of T cell activity within the TME. By comparing CAFs from CT26 tumors formed in immunocompetent or T cell-deficient athymic BALB/c mice, we observed that the presence of tumor-infiltrating T cells led to the upregulation of PD-L1 expression. The presence of T cells also led to increased expression of *Mrc2* (70, 71), which is associated with fibroblast activation, and *Cxcl12*, which is associated with the formation of an immunosuppressive and T cell-excluding TME (36, 41). Altogether, our data underscores that CAFs are highly plastic cells that are affected by cues from the TME and the observed gene expression profiles suggest that they can contribute to immunosuppression.

## Data availability statement

The datasets presented in this study can be found in online repositories. The names of the repository/repositories and accession number(s) can be found below: The data has been uploaded to GEO (accession number GSE245293).

## Ethics statement

The animal study was approved by the Danish Animal Experiment Council. The study was conducted in accordance with the local legislation and institutional requirements.

## Author contributions

MC: Formal Analysis, Investigation, Validation, Visualization, Writing – original draft, Writing – review & editing. M-LT: Formal Analysis, Investigation, Validation, Visualization, Writing – original draft, Writing – review & editing. AS: Formal Analysis, Investigation, Validation, Visualization, Writing – review & editing. DA: Formal Analysis, Investigation, Validation, Visualization, Writing – review & editing. AJ: Investigation, Validation, Visualization, Writing – review & editing, Formal Analysis. KB: Formal Analysis, Investigation, Validation, Visualization, Writing – review & editing. SK: Formal Analysis, Investigation, Validation, Visualization, Writing – review & editing. AR: Formal Analysis, Investigation, Validation, Visualization, Writing – review & editing. KF: Formal Analysis, Investigation, Validation, Visualization, Writing – review & editing. DK: Formal Analysis, Investigation, Validation, Visualization, Writing – review & editing. MD: Methodology, Project administration, Resources, Writing – review & editing. LG: Formal Analysis, Methodology, Resources, Validation, Visualization, Writing – review & editing. DM: Conceptualization, Funding acquisition, Methodology, Project administration, Resources, Supervision, Validation, Visualization, Writing – original draft, Writing – review & editing. HL: Formal analysis, Visualization, Writing – review & editing.
