# Supplementary material for: Dissecting tumor microenvironment heterogeneity in syngeneic mouse models: insights on cancer-associated fibroblast phenotypes shaped by infiltrating T cells
Source: Front Immunol. 2024 Jan 8;14:1320614. doi: 10.3389/fimmu.2023.1320614 (PMC10800379; doi:10.3389/fimmu.2023.1320614)
Supplement: Supplementary file 1 [file DataSheet_1.pdf]

Supplementary tables and figures

Table S1: RNAseq data whole tumors

Table S2: RNAseq data, isolated CAFs

Table S3: List of antibodies

Antibody	Cat. No.	Vendor	Panel
PE anti-human CD140a (PDGFRα)	323505	Biolegend	General
PE/Cy7 anti-mouse CD19	115520	Biolegend	Lymphoid
Brilliant Violet 421™ anti-mouse CD 274 (B7-H1, PD-L1)	124315	Biolegend	General
FITC anti-mouse CD31	102406	Biolegend	General
PE Rat 1gG2a, k isotype control	400508	Biolegend	General
FAP Biotinylated Antibody	BAF3715	R&D Systems/Bio-Techne	General
FITC anti-mouse CD11c	117306	Biolegend	Myeloid
Brilliant Violet 421™ anti-mouse CD4	100438	Biolegend	Lymphoid
APC/Cy7 anti-mouse NK1.1	108724	Biolegend	Lymphoid
APC anti-mouse CD8a	100712	Biolegend	Lymphoid
PE anti-mouse CD206 (MMR)	141705	Biolegend	Myeloid
FITC anti-mouse CD3	100204	Biolegend	Lymphoid
PE/Cy7 anti-mouse CD45	103114	Biolegend	General
Brilliant Violet 421™ anti-mouse CD103	121422	Biolegend	Myeloid
PE anti-mouse CD279 (PD-1)	109103	Biolegend	Lymphoid
PerCP/Cy5.5 anti-mouse CD25	101912	Biolegend	Lymphoid
APC/Cy7 anti-mouse Ly-6G	127623	Biolegend	Myeloid
APC anti-mouse F4/80	123116	Biolegend	Myeloid
PE/Cy7 anti-mouse CD11b	101215	Biolegend	Myeloid
PerCP/Cy5.5 anti-mouse Ly-6C	128011	Biolegend	Myeloid
Zombie Aqua™ Fixable Viability Kit	423102	Biolegend	All panels
APC Streptavidin	405207	Biolegend	General

Table S4: List of primers used for qRT-PCR

Gene	Primer name	Sequence (5' - 3')
Col1a1	Col1a1_fw	CTGACTGGAAGAGCGGAGAG
	Col1a1_rv	GACGGCTGAGTAGGGAACAC
Mrc2	Mrc2_fw	CCACAACAGCTGCTACTGGA
	Mrc2_rv	AGGGCTGCTGATGGCAAG
Acta2	Acta2_fw	CATCTTTCATTGGGATGGAGTCAG
	Acta2_rv	ACAGGACGTTGTTAGCATAGAGA
Il6	Il6_fw	GTCTTCTGGAGTACCATAGC
	Il6_rv	GTCAGATACCTGACAACAGG
Cxcl12	Cxcl12_fw	CGGTTCTTCGAGAGCCACAT
	Cxcl12_rv	GCCGTGCAACAATCTGAAGG
Tgfb1	Tgfb1_fw	AGCCCTGTATTCCGTCTCCT
	Tgfb1_rv	CTGCTGACCCCCACTGATAC
Arg1	Arg1_fw	CAGAAGAATGGAAGAGTCAG
	Arg1_rv	CAGATATGCAGGGAGTCACC
Cd274	Cd274_fw	TCACTTGCTACGGGCGTTT
	Cd274_rv	CCCAGTACACCACTAACGCA

Figure S1

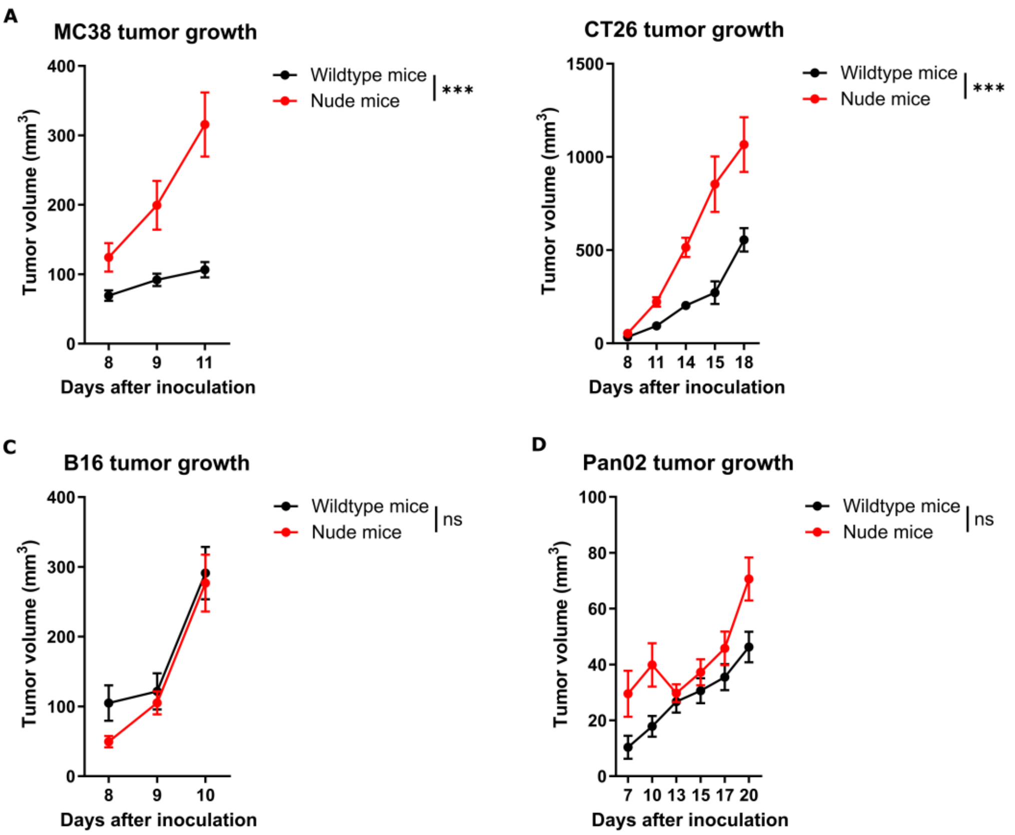

<u>**Figure S1:**</u>

Tumor growth of the syngeneic murine tumor models MC38, CT26, B16, and Pan02 in wildtype mice (black) and nude mice (red). A-D) Cancer cells were subcutaneously injected in the flank of C57BL/6 mice (MC38 (A), B16 (C), and Pan02 (D)) or BALB/c mice (CT26 (B)), n = 10-15. Error bars indicate the standard error of the mean (SEM). Statistical analysis was performed by two-way ANOVA with Bonferroni correction, *** = $p \leq 0.001$, ** = $p \leq 0.01$, * = $p \leq 0.05$not significant when $p > 0.05$.

Figure S2

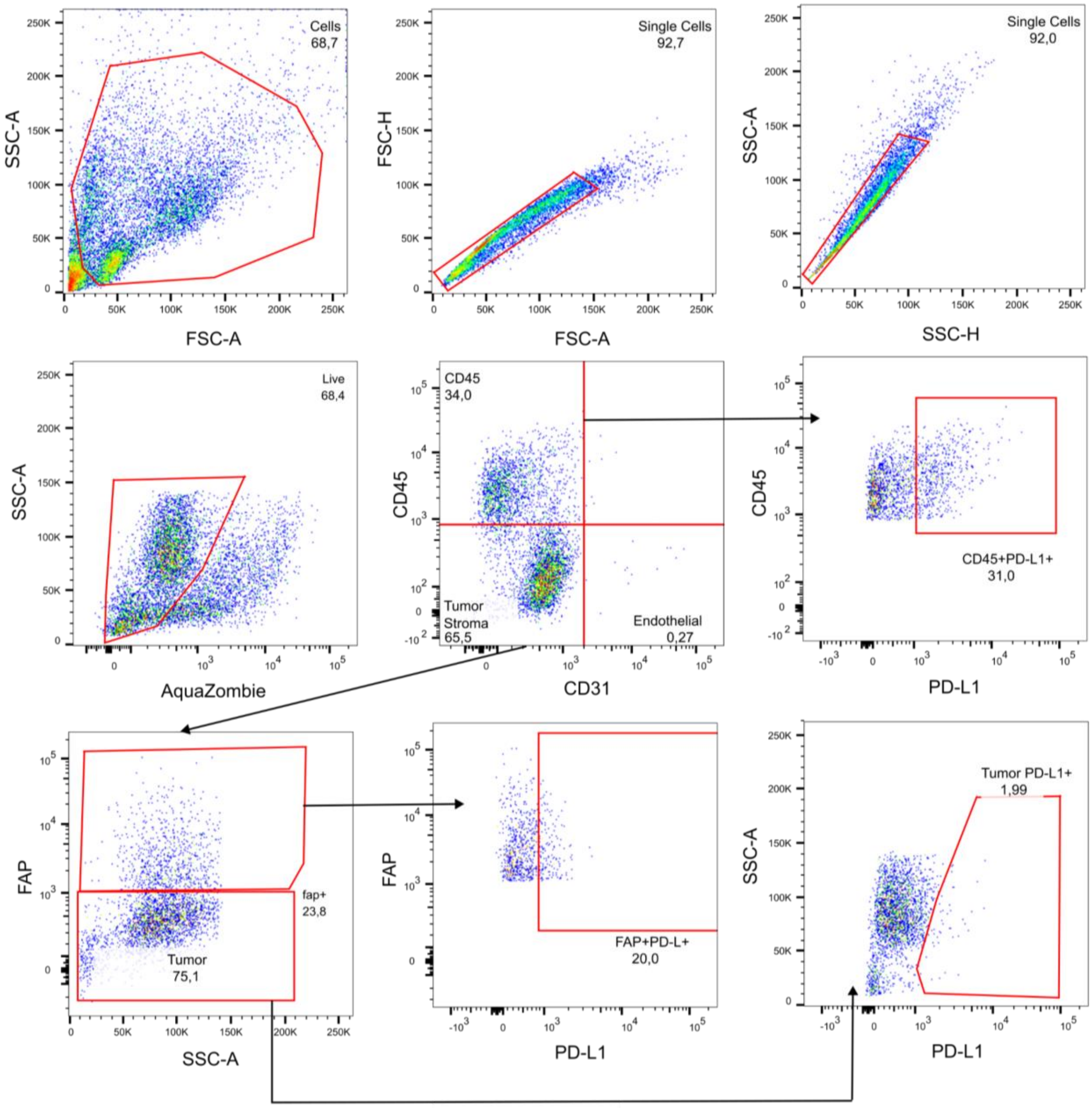

<u>**Figure S2:**</u>

Gating strategy for general panel. Representative plots from flow cytometry analysis showing the gating strategy for identification of the indicated cell populations from a single cell suspension of a LL2 tumor. After exclusion of doublets and dead cells (Zombie Aqua-negative), leukocytes ($CD45^+$) and endothelial cells ($CD31^+$) were identified. From the negative population, CAFs (FAP^+) and tumor cells ($CD45^-CD31^-FAP^-$) were identified. PD-L1 expression was assessed on $CD45^+$ cells, FAP^+ CAFs, and tumor cells.

Figure S3

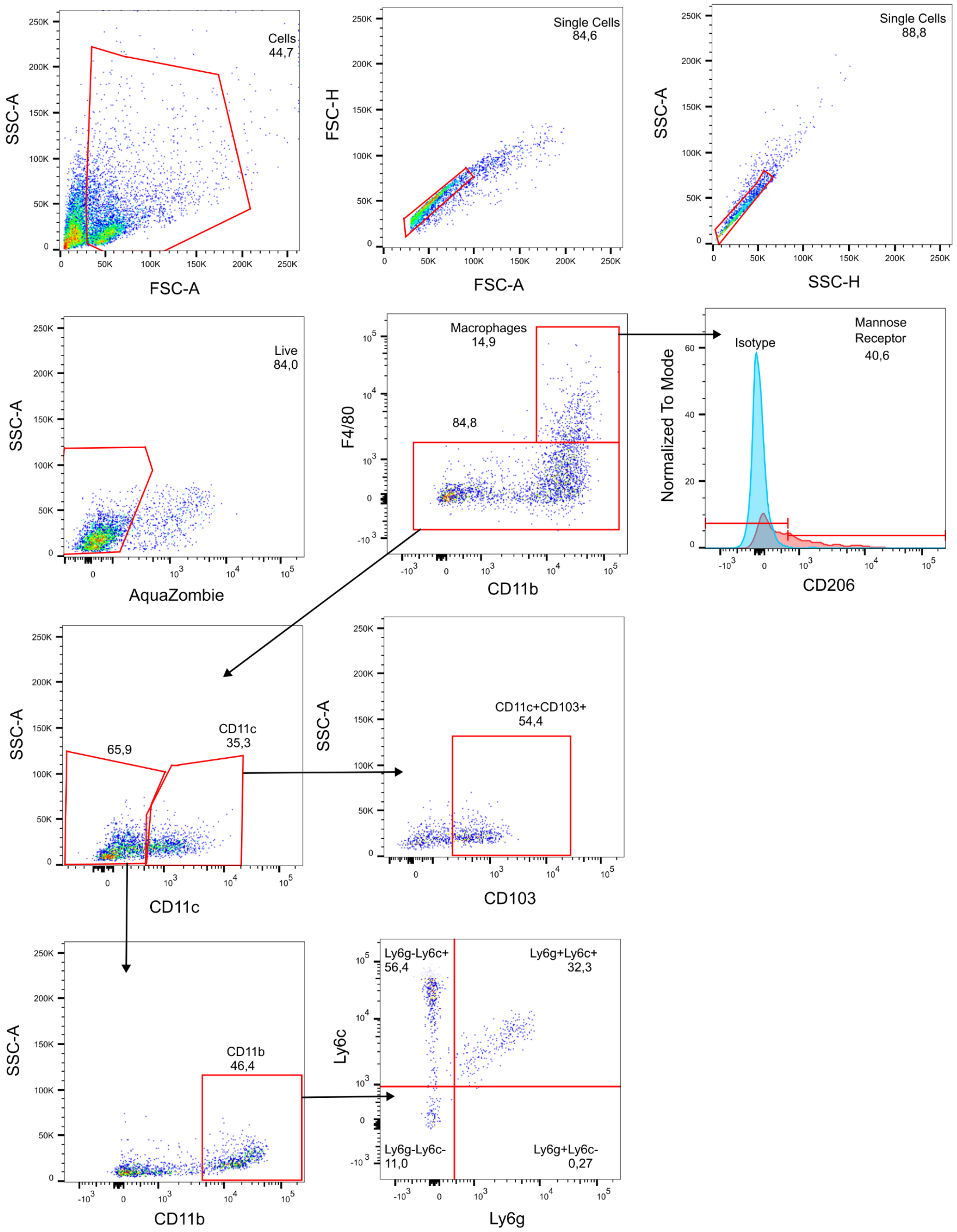

Figure S3:

Gating strategy for myeloid panel. Representative plots from flow cytometry analysis showing the gating strategy for identification of the indicated cell populations from a single cell suspension of a LL2 tumor. After exclusion of doublets and dead cells, TAMs (F4/80$^+$CD11b$^+$) were identified. Expression of the mannose receptor (CD206) was assessed on TAMs. Dendritic cells (F4/80$^-$CD11c$^+$) were identified and from this population CD103$^+$ dendritic cells were identified. M-MDSCs were identified as being CD11b$^+$F4/80$^-$Ly6C^{hi} and PMN-MDSCs as being CD11b$^+$F4/80$^-$Ly6C^{lo}Ly6G$^+$.

Figure S4

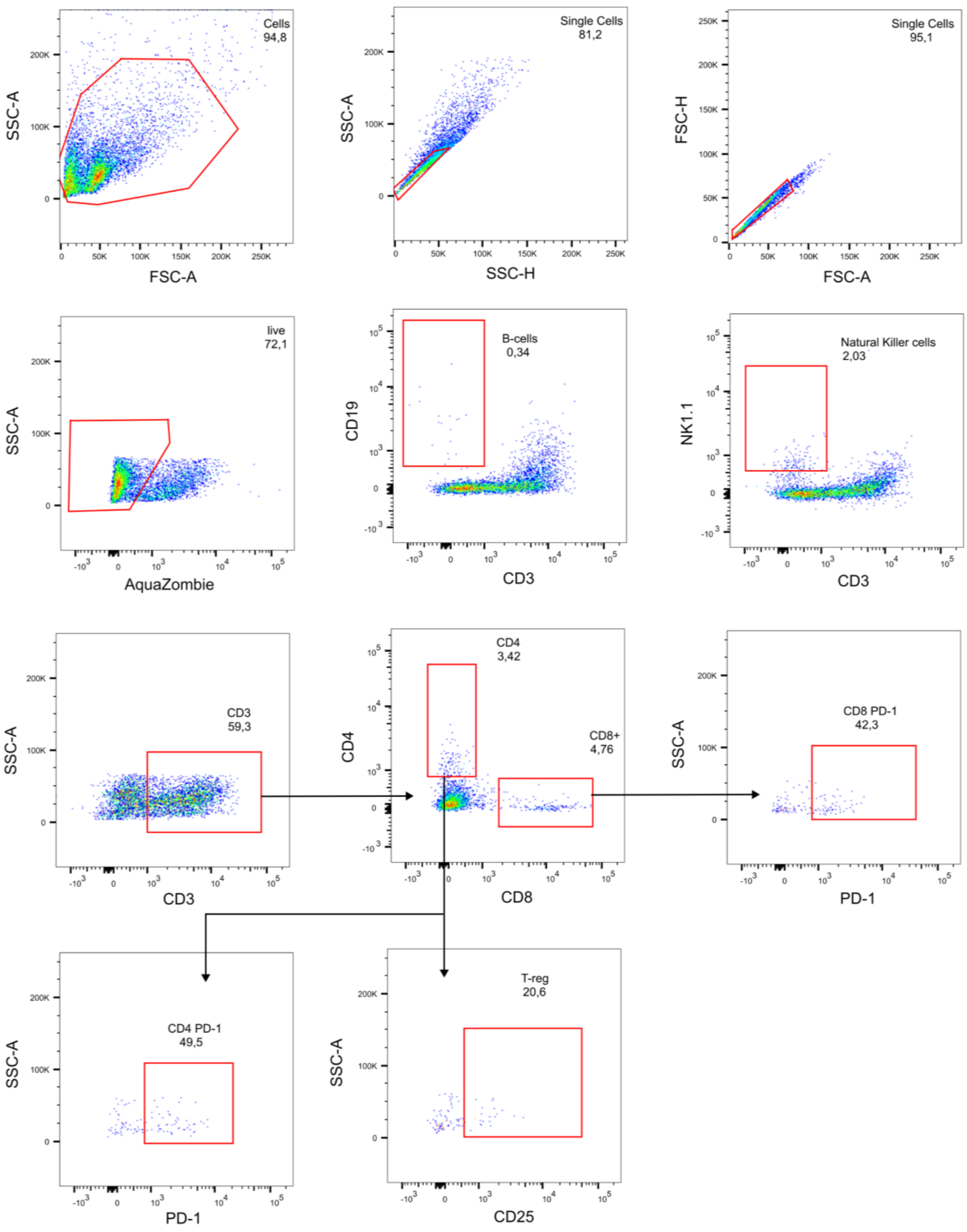

Figure S4:

Gating strategy for lymphoid panel. Representative plots from flow cytometry analysis showing the gating strategy for identification of the indicated cell populations from a single cell suspension of a LL2 tumor. After exclusion of doublets and dead cells, B cell and NK cells were identified as being $CD3^-CD19^+$ and $CD3^-NK1.1^+$, respectively. From the $CD3^+$ population, $CD4^+$ and $CD8^+$ T cells were identified. Within the $CD4^+$ populations, T_{regs} ($CD4^+CD25^+$) were identified. Expression of PD-1 was assessed on $CD4^+$ and $CD8^+$ T cells.

Figure S5

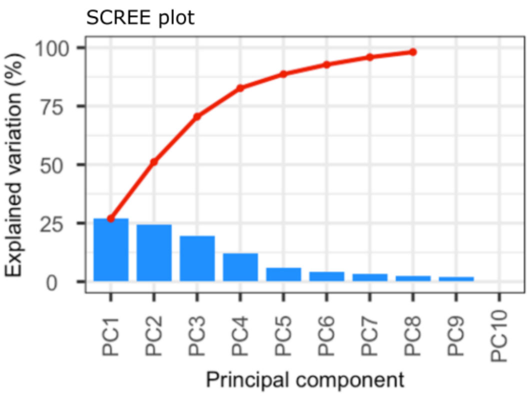

Figure S5:

SCREE plot describing the variability between samples. The SCREE plot analysis was based on sequenced RNA from FACS-sorted FAP^{+} CAFs.

Figure S6

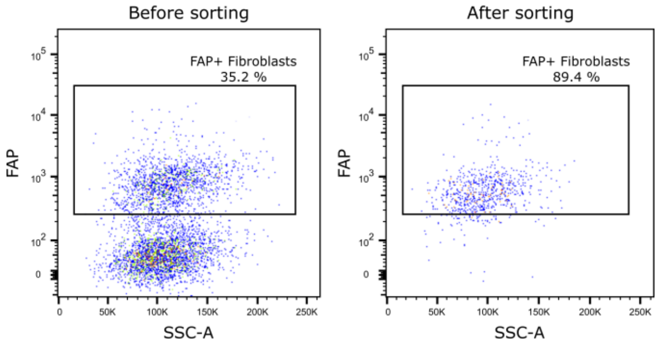

<u>Figure S6:</u>

Sorting efficiency of FAP$^+$ fibroblasts. Representative dot plots from FACS showing the gating and efficiency of sorting. Sorting was performed after enrichment of CD45$^-$ cells from a single-cell suspension of tumors. Prior to gating for FAP, debris, doublets, and dead cells were excluded.
